# Association of admission serum triglyceride levels with intensive care unit hospitalization rates in acute pancreatitis patients: A retrospective study

**DOI:** 10.1097/MD.0000000000038265

**Published:** 2024-05-24

**Authors:** Shuaiyong Wen, Yu Zhang, Guijie Zhao, Zhengwei Tu, Kun Zhang, Yunfeng Cui

**Affiliations:** a Tianjin Medical University, Tianjin, China; b Tianjin Key Laboratory of Acute Abdomen Disease Associated Organ Injury and ITCWM Repair, Tianjin NanKai Hospital, Tianjin, China; c Department of Surgery, Tianjin Nankai Hospital, Nankai Clinical School of Medicine, Tianjin Medical University, Tianjin, China.

**Keywords:** cohort, hypertriglyceridemia, ICU, pancreatitis

## Abstract

Acute pancreatitis (AP) is a complex and unpredictable condition, of which hypertriglyceridemia (HTG) is the third most prevalent cause. This study aimed to conduct a retrospective analysis of clinical data from hospitalized AP patients to uncover a potential correlation between triglyceride (TG) levels and the necessity for intensive care unit (ICU) admission. This retrospective cohort study utilized the Medical Information Mart for Intensive Care IV 2.2 (MIMIC-IV) critical care dataset, incorporating data from 698 patients with hypertriglyceridemic acute pancreatitis (HTG-AP). The analysis employed the RCS model along with univariate and multivariate logistic regression methods to affirm the association between triglyceride levels and ICU admission. Subgroup analysis was performed to investigate specific populations. The study included 698 patients with AP, 42.41% of whom experienced HTG during hospitalization. RCS analysis revealed a linear association between TG levels and risk of ICU admission (*p* for nonlinear = .219, *p* for overall = .009). Multivariate logistic regression analysis indicated an increased risk of ICU admission in the TG range of 1.7–5.65 mmol/L (aOR = 1.83, 95% CI 1.12–2.99, *P* = .015) and TG >11.3 mmol/L (aOR = 5.69, 95% CI 2.36–13.74, *P* < .001) compared to the normal group. Similar results were observed across the various subgroups. As triglyceride levels increased, there was a corresponding increase in ICU admissions. Patients within the 1.7 to 5.65 mmol/L and > 11.3 mmol/L triglyceride groups exhibited higher rates of ICU admissions. Moreover, we observed a higher risk of ICU hospitalization even with mild TG elevation.

## 1. Introduction

Acute pancreatitis (AP) is a prevalent gastrointestinal disorder that often leads to hospital admission in the United States. This condition is characterized by its complexity and unpredictability, with approximately 1 in 5 patients experiencing progression to severe pancreatitis. The mortality rate for individuals with severe pancreatitis is estimated to be approximately 20%.^[1,2]^ Hypertriglyceridemia (HTG) is the third primary cause of acute pancreatitis, constituting 4% to 10% of cases, trailing behind gallstones (up to 60%) and alcohol (30%).^[3]^ Primary HTG is triggered by genetic and environmental factors, including a high-fat, high-carbohydrate diet as well as a disruption in triglyceride (TG) synthesis and metabolism resulting from a sedentary lifestyle.^[4]^ Secondary HTG often emerges due to undiagnosed or uncontrolled diabetes, obesity, metabolic syndrome, alcohol consumption, pregnancy, cholelithiasis, and the use of medications such as tamoxifen, estrogens, and atypical antipsychotics.^[3,4]^ The incidence of hypertriglyceridemic acute pancreatitis (HTG-AP) is projected to increase because of the increasing prevalence of overweight, obesity, and overeating habits.^[5]^

According to expert consensus, the vast majority agree that AP cases with TG levels ≥ 5.6 mmol/L should be suspected as HTG-AP, while cases with TG levels ≥ 11.3 mmol/L are confirmed to be HTG-AP.^[6]^ The intricate pathological mechanism underlying TG and AP involves pancreatic lipase hydrolysis of triglycerides, excessive formation of free fatty acids resulting in inflammatory changes and capillary damage, and potential factors such as hyperviscosity and ischemia, which play crucial roles.^[7]^

While HTG is not the main cause of acute pancreatitis, it has been observed that the risk of developing a severe (moderately severe and severe) condition is the highest in HTG-AP.^[8]^ TG levels were associated with the number of hospital days and ICU days in the 24h and/or 48h groups.^[9]^ A study revealed that patients admitted to the ICU exhibited higher mean TG concentrations than those not admitted to the ICU, and HTG-AP displayed a higher rate of ICU hospitalization than pancreatitis, attributed to biliary factors.^[10]^ Elevated blood triglyceride concentrations are associated with increased rates of ICU hospitalization. However, the precise nature of this relationship requires further investigation. Therefore, this study aimed to retrospectively analyze clinical data from hospitalized patients with AP to determine the potential correlation between TG levels and the need for ICU admission.

## 2. Methods

### 2.1. Study design and data source

The information presented in this investigation was extracted from the Medical Information Mart for Intensive Care IV (MIMIC-IV, version 2.2), an openly available database dedicated to critical care. It includes extensive and top-notch information on individuals hospitalized in intensive care units (ICUs) at the Beth Israel Deaconess Medical Center from 2008 to 2019.^[11–13]^ Author Shuaiyong Wen accessed the database after successfully completing an online course and passing the National Institutes of Protecting Human Research Participants Exam (certification number: 55403300).

To protect patient privacy, all personally identifiable information was anonymized. Given the complete de-identification of patient records in the MIMIC-IV database, the institutional review board of Beth Israel Deaconess Medical Center deemed individual patient consent unnecessary.

### 2.2. Inclusion and exclusion criteria

**Figure 1.**
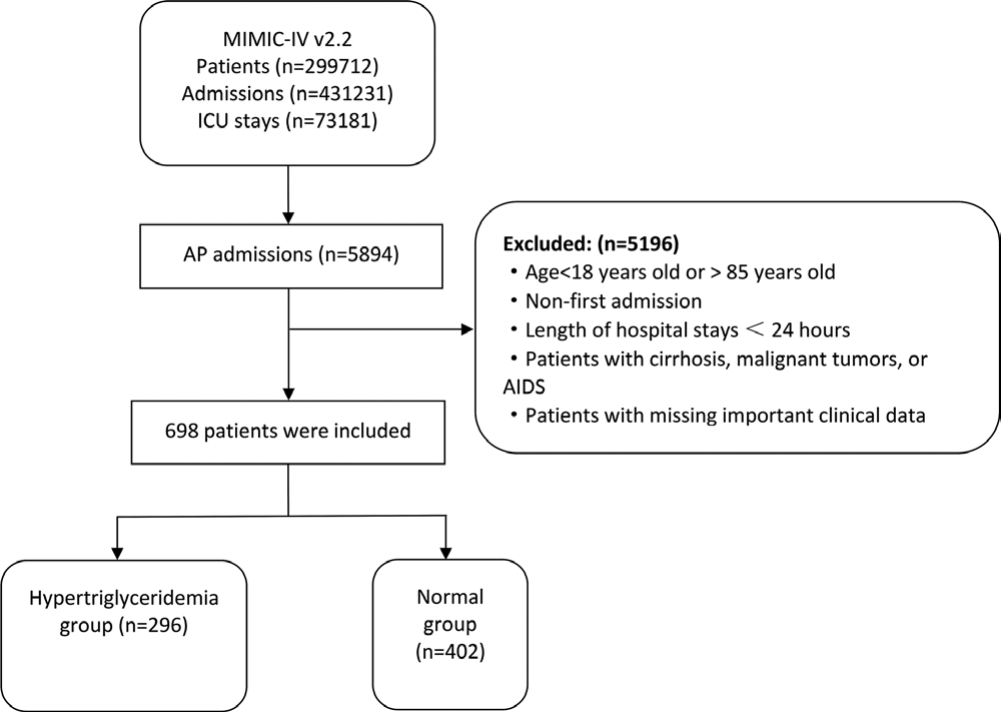
Flow chart.

Using Structured Query Language, clinical data were obtained from the MIMIC-IV database for patients meeting the defined inclusion and exclusion criteria. The inclusion criteria included patients diagnosed with AP, as indicated by the 9^th^ Revision of the International Classification of Diseases (ICD-9) code 577.0, and the 10^th^ Revision (ICD-10) codes K85-K85.92. A total of 5894 patients meeting these criteria were included in the study. The exclusion criteria were as follows: patients under 18 or over 85 at the initial admission; multiple admissions for acute pancreatitis, retaining only the data from the first admission; hospital stay <24 hours; diagnosis of cirrhosis, malignant tumors, or acquired immunodeficiency syndrome (AIDS); and patients with missing essential clinical data, such as TG. The study included 698 patients (Fig. 1).

### 2.3. Data extraction and definitions

We extracted the following outcome variables: age, gender, ethnicity, height, weight, comorbidities [hypertension, diabetes, coronary heart disease (CHD), acute kidney injury (AKI), respiratory failure (RF), heart failure (HF), atrial fibrillation (AF), obesity], laboratory parameters [TG, TG max, TG min, total cholesterol (TC), high-density lipoprotein (HDL), low-density lipoprotein (LDL), lipase, amylase, total bilirubin (TBIL), aspartate transaminase (AST), alanine aminotransferase (ALT), alkaline phosphatase (ALP), albumin, red blood cell (RBC), white blood cell (WBC), red blood cell distribution width (RDW), platelets, hemoglobin, glucose, blood urea nitrogen (BUN), creatinine, C-reactive protein (CRP), sodium, potassium, calcium, platelet, fibrinogen, prothrombin time (PT)], abdominal necrosis, pancreatic pseudocyst, ICU admission, ICU stays, hospital stays, recurrent acute pancreatitis (RAP), and 30-day mortality.

All baseline data were obtained from patients’ first hospital admission measurements. Based on ethnicity, patients were divided into white and nonwhite groups. The Adult Treatment Panel III guidelines of the National Cholesterol Education Program and the Endocrine Society all consider normal TG to be < 1.7 mmol/L, and values above that are considered HTG.^[14,15]^ Patients with AP were categorized into HTG and normal groups based on whether their TG levels exceeded 1.7 mmol/L. Furthermore, for a more in-depth analysis of the correlation between TG levels at admission and ICU admission, we further subdivided the patient groups. In general, serum TG ≥ 11.3 mmol/L is considered a condition that induces HTG-AP, but there is no clear threshold.^[16]^ Half of the studies included in a meta-analysis used 5.65 mmol/L as the minimum value for the diagnosis of HTG-AP.^[17]^ To investigate the effect of different levels of TG on the outcome of patients with AP, we categorized TG into 4 groups based on a previous study^[18]^: Normal group: TG < 1.7 mmol/L; Mildly elevated group: 1.7 ≤ TG < 5.65 mmol/L; Moderately elevated group: 5.65 mmol/L ≤ TG < 11.3 mmol/L; Severely elevated group: TG ≥ 11.3 mmol/L.

### 2.4. Missing data and outliers management

To mitigate bias, variables with > 20% missing values, including height, weight, TC, HDL, LDL, amylase, albumin, CRP, and fibrinogen, were excluded from the analysis.

Variables with missing values between 5% and 20% (total bilirubin, AST, ALT, ALP, PT, and lipase) were subjected to multiple imputations (MI).^[19]^ This method was employed to choose the most suitable dataset for filling in missing values. On the other hand, variables with missing values <5% (RBC, WBC, RDW, PLT, hemoglobin, glucose, BUN, creatinine, sodium, potassium, and calcium) were replaced with the mean value of that variable. Abnormal values in the variables are addressed using the winsorize method, specifically the winsor2 command, with cutoff points set at 1% and 99%. This technique minimizes the influence of extreme values in the analysis. STATA software (version 17) was employed to manage missing and abnormal data, ensuring a thorough and systematic approach to data processing.

### 2.5. Statistical analysis

Initial normality testing was conducted for the continuous variables. Normally distributed variables are reported as mean ± standard deviation, whereas non-normally distributed variables are presented as median and interquartile range. Categorical variables were presented as counts (percentages). *P* values for categorical variables were analyzed using chi-square tests, and for continuous variables, the Kruskal-Wallis test was employed.

To offer a more intuitive representation of the dose-response relationship between continuous variables and outcomes, the restricted cubic spline (RCS) function was implemented. In this study, the RCS model was used to confirm the association between TG level and ICU admission. Univariate and multivariate logistic regression analyses were used to assess the association between TG levels and ICU admission. Adjusted odds ratios (aORs) and the corresponding 95% confidence intervals (CIs) were calculated. Multivariate logistic regression analysis involved the construction of 3 models: Model A, Model B, and Model C. Model A included 3 demographic variables (age, sex, and ethnicity). Model B was expanded on Model A by adding 8 comorbidity variables (hypertension, diabetes, CHD, AKI, RF, HF, AF, and obesity). Model C further extended Model B by including 17 laboratory test variables (lipase, TBIL, AST, ALT, ALP, RBC, WBC, RDW, platelets, hemoglobin, glucose, BUN, creatinine, sodium, potassium, calcium, and PT). Finally, subgroup analysis was conducted to explore the potential effects of TG in different subgroups, including age, sex, ethnicity, hypertension, diabetes, CHD, AKI, and obesity.

Statistical analyses were performed using SPSS software (version 24.0) and R Studio software (version 1.7.7). Statistical significance was determined at *P* < .05.

## 3. Results

### 3.1. Demographic and clinical characteristics at baseline

A total of 698 patients with AP were included in the study based on predefined inclusion and exclusion criteria. Among them, 296 (42.41%) patients exhibited HTG (TG ≥ 1.7 mmol/L) during hospitalization, whereas the remaining 402 (57.59%) did not. The baseline characteristics of the HTG and control groups are detailed in Table 1, including age, sex, ethnicity, comorbidities, and laboratory parameters. Demographically, the HTG group had a higher likelihood of being female and elderly. Additionally, the HTG group had a higher prevalence of diabetes (40.54% vs 23.88%), acute kidney injury (AKI, 47.30% vs 25.37%), and respiratory failure (RF, 39.19% vs 16.42%) than the control group.

**Table 1:** Demographical characteristics and clinical data of the patients.

Variables	Hypertriglyceridemia group (n = 296)	Normal group (n = 402)	*P*
Age, (yr)	56.17 (40.64, 67.86)	49.32 (40.12, 60.05)	**<.001**
Female, n (%)	205 (69.26)	211 (52.49)	**<.001**
Ethnicity, n (%)			.259
Nonwhite	101 (34.12)	121 (30.10)	
White	195 (65.88)	281 (69.90)	
Comorbidities			
Hypertension, n (%)	102 (34.46)	142 (35.32)	.813
Diabetes, n (%)	120 (40.54)	96 (23.88)	**<.001**
CHD, n (%)	40 (13.51)	53 (13.18)	.899
AKI, n (%)	140 (47.30)	102 (25.37)	**<.001**
RF, n (%)	116 (39.19)	66 (16.42)	**<.001**
HF, n (%)	40 (13.51)	38 (9.45)	.092
AF, n (%)	29 (9.80)	52 (12.64)	.201
Obesity, n (%)	59 (19.93)	59 (14.68)	.067
Laboratory parameters			
TG, (mmol/L)	2.98 (2.17, 5.30)	1.08 (0.86, 1.33)	**<.001**
TG max, (mmol/L)	3.17 (2.24, 7.25)	1.10 (0.87, 1.40)	**<.001**
TG min, (mmol/L)	2.45 (1.93, 3.48)	1.05 (0.84, 1.33)	**<.001**
Lipase, (IU/L)	172.50 (68.50, 636.50)	172.50 (68.50, 636.00)	.592
TBIL, (mg/mL)	0.80 (0.50, 1.70)	0.72 (0.50, 1.30)	.245
AST, (IU/L)	48.50 (27.00, 111.00)	36.50 (20.00, 92.00)	**.006**
ALT, (IU/L)	38.00 (21.00, 88.00)	36.09 (17.00, 117.00)	.858
ALP, (IU/L)	82.50 (58.50, 126.50)	88.50 (63.00, 137.00)	.125
RBC, (m/μL)	4.06 (3.58, 4.53)	3.90 (3.43, 4.49)	**.028**
WBC, (K/μL)	10.80 (7.45, 15.30)	10.30 (7.00, 14.90)	.330
RDW, (%)	13.85 (13.15, 14.80)	13.70 (12.90, 14.50)	**.009**
Platelets, (K/μL)	208.50 (160.00, 283.50)	235.50 (179.00, 293.00)	**.003**
Hemoglobin, (g/dL)	12.20 (10.80, 13.60)	12.12 (10.50, 13.63)	.674
Glucose, (mg/dL)	135.00 (105.00, 196.50)	105.00 (88.00, 130.00)	**<.001**
BUN, (mg/dL)	15.00 (10.00, 27.00)	12.00 (8.00, 18.85)	**<.001**
Creatinine, (mg/dL)	1.00 (0.70, 1.55)	0.80 (0.60, 1.00)	**<.001**
Sodium, (mEq/L)	139.00 (137.00, 141.00)	138.00 (135.00, 141.00)	**<.001**
Potassium, (mEq/L)	4.00 (3.70, 4.40)	3.95 (3.60, 4.20)	**.004**
Calcium, (mg/dL)	8.26 (7.40, 8.80)	8.50 (8.10, 9.00)	**<.001**
PT, (s)	13.09 (11.94, 14.50)	13.70 (12.50, 15.40)	**<.001**
Abdominal necrosis, n (%)	38 (12.84)	32 (7.96)	**.034**
Pancreatic pseudocyst, n (%)	59 (19.93)	83 (20.65)	.817
ICU admission, n (%)	199 (67.23)	141 (35.07)	**<.001**
ICU stays, (d)	4.33 (2.06, 10.61)	2.92 (1.38, 7.17)	**<.001**
Hospital stays, (d)	9.92 (4.53, 21.56)	6.08 (3.42, 11.71)	**<.001**
RAP, n (%)	82 (27.70)	105 (26.12)	.641
30-day mortality, n (%)	11 (3.72)	5 (1.24)	**.031**

Bold values are statistically significant *P* < 0.05.

AF = atrial fibrillation, AKI = acute kidney injury, ALP = alkaline phosphatase, ALT = alanine aminotransferase, AST = aspartate transaminase, BUN = blood urea nitrogen, CHD = coronary heart disease, HF = heart failure, ICU = intensive care unit, PAP = recurrent acute pancreatitis, PT = prothrombin time, RBC = red blood cell, RDW = red blood cell, RF = respiratory failure, TBIL = total bilirubin, TG = triglyceride, WBC = white blood cell.

In terms of laboratory parameters, the HTG group exhibited higher levels of AST, RBC, RDW, glucose, BUN, creatinine, sodium, and potassium and lower platelet and calcium levels. The hyperlipidemia group also showed a higher incidence of abdominal necrosis (12.84% vs 7.96%, *P* = .034). Furthermore, the HTG group had a higher rate of ICU admission (67.23% vs 35.07%, *P* < .001), along with prolonged ICU and hospital stay. Additionally, patients in the hyperbilirubinemia group had a higher 30-day mortality rate (3.72% vs 1.24%, *P* = .031) than those in the normal group.

### 3.2. Association of TG with clinical outcomes

**Figure 2.**
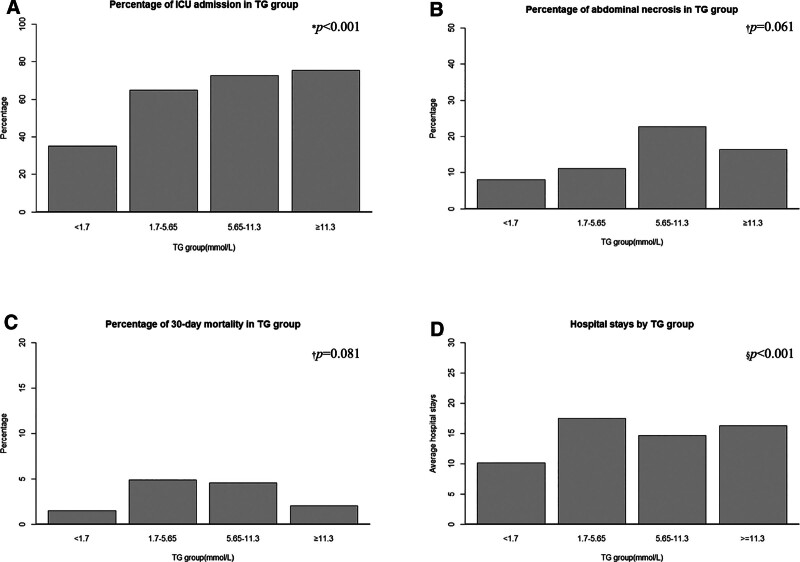
The histogram of relationship between TG groups and clinical outcomes. (A), TG groups and ICU admission; (B), TG groups and abdominal necrosis; (C), TG groups and 30-day mortality; (D), TG groups and average hospital stays. *Chi-square test; †Fisher exact test; §Kruskal-Wallis test.

**Figure 3.**
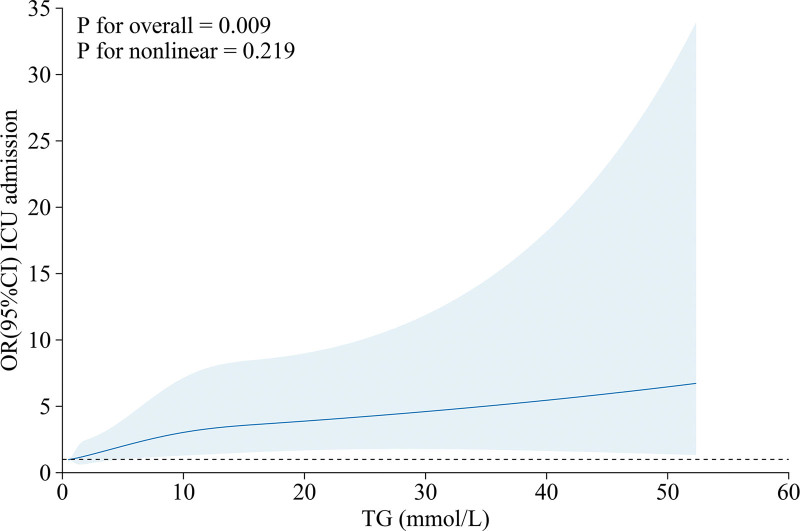
Illustration of restricted cubic spline logistic regression models depicting the relationship between TG levels and ICU admission. The solid blue line illustrates the odds ratio (OR) as a function of TG, adjusted for age, ethnicity, diabetes, AKI, CHD, RF, HF, AF, obesity, TBIL, AST, ALT, WBC, RBC, RDW, platelets, hemoglobin, glucose, BUN, creatinine, potassium, calcium, and PT (Variable selection details can be found in Table 2). AF = atrial fibrillation, AKI = acute kidney injury, ALT = alanine aminotransferase, AST = aspartate transaminase, BUN = blood urea nitrogen, CHD = coronary heart disease, HF = heart failure, PT = prothrombin time.

To better scrutinize the association between TG and clinical outcomes, we stratified TG into 4 groups. The histogram of ICU admission, abdominal necrosis, 30-day mortality, and average hospital stay in each TG group was plotted, and the relationship between TG and different clinical outcomes was preliminarily observed (Fig. 2). All high-TG groups had higher rates of ICU admission, abdominal necrosis, 30-day mortality, and longer hospital stay than the normal group. Grouped according to whether they were admitted to the ICU or not and statistically described (Table 2). RCS analysis was subsequently performed and adjusted for variables with *P *< .05. RCS analysis revealed a linear association between TG levels and the risk of ICU admission (*p* for nonlinear = .219, *p* for overall = .009; Fig. 3).

**Table 2:** Population demographic characteristics and clinical data grouped based on ICU admissions.

Variables	NON-ICU (n = 358)	ICU (n = 340)	*P*
Age, (yr)	51.86 (38.20, 62.42)	54.09 (43.49, 66.43)	**.006**
Female, n (%)	208 (58.10)	208 (61.18)	.408
Ethnicity, n (%)			**<.001**
Nonwhite	267 (74.58)	209 (61.47)	
White	91 (25.42)	131 (38.53)	
Comorbidities			
Hypertension, n (%)	119 (33.24)	125 (36.76)	.329
Diabetes, n (%)	79 (22.07)	137 (40.29)	**<.001**
CHD, n (%)	35 (9.78)	58 (17.06)	**.005**
AKI, n (%)	58 (16.20)	184 (54.12)	**<.001**
RF, n (%)	14 (3.91)	168 (49.41)	**<.001**
HF, n (%)	19 (5.31)	59 (17.35)	**<.001**
AF, n (%)	20 (5.59)	61 (17.94)	**<.001**
Obesity, n (%)	46 (12.85)	72 (21.18)	**.003**
Laboratory parameters			
TG, (mmol/L)	1.27 (0.91, 1.78)	2.04 (1.17, 3.65)	**<.001**
TG max, (mmol/L)	1.29 (0.92, 1.90)	2.15 (1.24, 4.33)	**<.001**
TG min, (mmol/L)	1.24 (0.91, 1.74)	1.74 (1.08, 2.61)	**<.001**
Lipase, (IU/L)	176.50 (63.00, 624.75)	202.61 (77.50, 723.50)	.236
TBIL, (mg/mL)	0.70 (0.40, 1.20)	0.90 (0.50, 1.80)	**<.001**
AST, (IU/L)	31.33 (20.00, 71.75)	55.00 (30.00, 131.25)	**<.001**
ALT, (IU/L)	33.00 (17.00, 87.50)	42.50 (21.75, 127.00)	**.003**
ALP, (IU/L)	85.00 (62.00, 129.00)	86.59 (62.00, 132.25)	.602
RBC, (m/μL)	4.06 (3.67, 4.51)	3.88 (3.37, 4.50)	**.009**
WBC, (K/μL)	9.40 (6.50, 12.88)	12.35 (8.45, 16.82)	**<.001**
RDW, (%)	13.40 (12.70, 14.03)	14.10 (13.40, 15.10)	**<.001**
Platelets, (K/μL)	236.50 (186.00, 295.75)	207.00 (156.00, 282.25)	**<.001**
Hemoglobin, (g/dL)	12.30 (10.90, 13.70)	12.05 (10.40, 13.43)	**.040**
Glucose, (mg/dL)	104.00 (87.00, 128.00)	131.00 (103.75, 193.25)	**<.001**
BUN, (mg/dL)	11.00 (8.00, 16.00)	17.50 (11.00, 30.00)	**<.001**
Creatinine, (mg/dL)	0.80 (0.60, 1.00)	1.00 (0.70, 1.60)	**<.001**
Sodium, (mEq/L)	139.00 (137.00, 141.00)	138.00 (135.00, 141.00)	.442
Potassium, (mEq/L)	4.00 (3.70, 4.20)	4.00 (3.70, 4.43)	**.007**
Calcium, (mg/dL)	8.60 (8.20, 9.00)	8.10 (7.40, 8.72)	**<.001**
PT, (s)	13.4 (12.0, 14.7)	13.6 (12.3, 15.4)	.069

Bold values are statistically significant *P* < 0.05.

AF = Atrial Fibrillation, AKI = Acute Kidney Injury, ALP = Alkaline Phosphatase, ALT = Alanine Aminotransferase, AST = Aspartate Transaminase, BUN = Blood Urea Nitrogen, CHD = Coronary Heart Disease, HF = Heart Failure, PT = Prothrombin Time, RBC = Red Blood Cell, RDW = Red Blood Cell Distribution Width, RF = Respiratory Failure, TBIL = Total Bilirubin, TG = Triglyceride, WBC = White Blood Cell.

### 3.3. Association of TG levels with ICU admission

Logistic regression analysis was used to examine the correlation between TG level and ICU admission (Table 3). In the univariate analysis, there was an increased risk of ICU admission in TG categories of 1.7 to 5.65 mmol/L (OR = 3.42, 95%CI 2.43–4.82), 5.56–11.3 mmol/L (OR = 4.94, 95%CI 1.89–12.90), and >11.3 mmol/L (OR = 5.71, 95%CI 2.88–11.29) compared to the normal group.

**Table 3:** Relative risk of ICU admission was calculated according to the TG level in different groups.

Variables	N	Univariate analysis		Model A		Model B		Model C	
		OR (95% CI)	*P*	aOR (95% CI)	*P*	aOR (95% CI)	*P*	aOR (95% CI)	*P*
TG, (mmol/L)	698	1.07 (1.03–1.10)	**<.001**	1.07 (1.04–1.11)	**<.001**	1.06 (1.03–1.09)	**<.001**	1.05 (1.02–1.09)	**.005**
TG < 1.7	402	1.00 (Ref)		1.00 (Ref)		1.00 (Ref)		1.00 (Ref)	
TG 1.7 to 5.65	225	3.42 (2.43–4.82)	**<.001**	3.97 (2.77–5.71)	**<.001**	2.22 (1.44–3.44)	**<.001**	1.83 (1.12–2.99)	**.015**
TG 5.65 to 11.3	22	4.94 (1.89–12.90)	**.001**	5.54 (2.05–14.97)	**<.001**	2.80 (0.81–9.72)	.105	1.55 (0.38–6.26)	.538
TG > 11.3	49	5.71 (2.88–11.29)	**<.001**	7.57 (3.72–15.37)	**<.001**	6.50 (2.97–14.20)	**<.001**	5.69 (2.36–13.74)	**<.001**

Bold values are statistically significant *P* < 0.05.

Adjusted covariates included model A (three variables in demographic information: age, sex, and ethnicity), model B (model A plus 8 variables in comorbidity information: hypertension, diabetes, CHD, AKI, RF, HF, AF, and obesity), and model C (model B plus 17 variables in laboratory test information: lipase, TBIL, AST, ALT, ALP, RBC, WBC, RDW, platelets, hemoglobin, glucose, BUN, creatinine, sodium, potassium, calcium, and PT).

In the multivariate logistic regressions for model A, there was an increased risk of ICU admission in the TG range of 1.7 to 5.65 mmol/L (aOR = 3.97, 95% CI 2.77–5.71, *P* < .001), 5.56–11.3 mmol/L (aOR = 5.54, 95% CI 2.05–14.97, *P* < .001), and >11.3 mmol/L (aOR = 7.57, 95% CI 3.72–15.37) compared to the normal group. In multivariate logistic regressions for model B, there was an increased risk of ICU admission in the TG range of 1.7–5.65 mmol/L (aOR = 2.22, 95% CI 1.44–3.44, *P < *.001), and >11.3 mmol/L (aOR = 6.50, 95% CI 2.97–14.20, *P* < .001) compared to the normal group. In multivariate logistic regressions for model C, adjusted for all variables, there was an increased risk of ICU admission in the TG range of 1.7 to 5.65 mmol/L (aOR = 1.83, 95% CI 1.12–2.99, *P* = .015), and >11.3 mmol/L (aOR = 5.69, 95% CI 2.36–13.74, *P* < .001) compared to the normal group.

### 3.4. Stratified analyses

**Figure 4.**
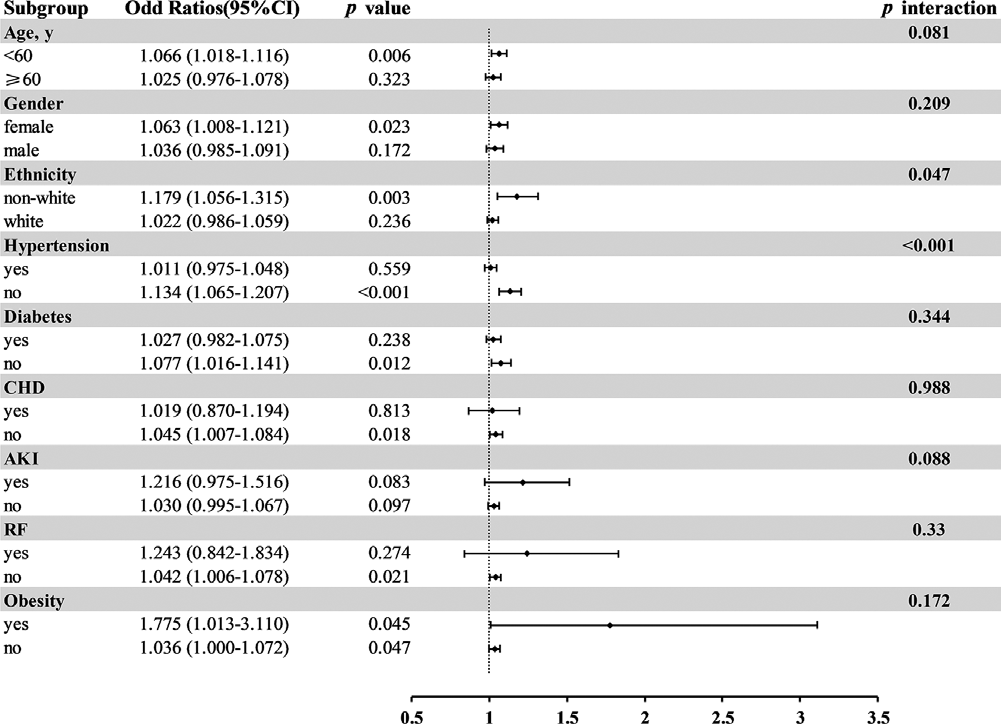
Subgroup analyses for the association of TG with ICU admission. CHD = coronary heart disease, AKI = acute kidney injury, RF = respiratory failure.

Figure 4 illustrates whether the correlation between TG levels and ICU admission in patients with AP remained stable across the subgroups. A stratified analysis was performed for age, sex, ethnicity, hypertension, diabetes, CHD, AKI, RF, and obesity. Overall, the relationship between TG and ICU admission was consistent across most subpopulations. However, a higher prevalence of increased ICU admission was observed in non-Caucasian patients [OR (95% CI), 1.179 (1.056, 1.315), *p* for interaction = 0.047]. Additionally, patients without hypertension had a higher ICU admission rate [OR, 1.134 (95% CI) 1.134 (1.065, 1.207); *P* < .001].

## 4. Discussion

In this retrospective study, we incorporated clinical data from the MIMIC-IV database, specifically examining 698 hospitalized patients diagnosed with AP. We utilized RCS dose-response curve analysis, along with univariate and multivariate logistic regression analyses, to explore the relationship between TG levels and the probability of ICU admission. Our findings revealed a significant linear relationship between TG levels and the probability of ICU admission in patients with AP. Additionally, our study revealed associations between HTG and various clinical outcomes, including abdominal necrosis (*P* = .034), length of ICU stay (*P* < .001), length of hospital stay (*P* < .001), and 30-day mortality (*P* = .031), which is consistent with previous studies.^[18,20–22]^

Multivariate logistic regression analysis showed that even slight elevations in TG levels were linked to a heightened risk of ICU hospitalization, further supporting previous investigations. Moreover, significantly elevated TG levels demonstrated a notably increased risk of ICU hospitalization. Interestingly, the group with moderately elevated lipid levels did not yield favorable results in models B and C.

In a comprehensive study encompassing 1233 patients with AP, all major adverse clinical outcomes (pancreatic necrosis, multiple organ dysfunction syndrome, organ failure, and mortality) exhibited a significant and progressive increase, directly correlating with admission triglyceride levels.^[18]^ In a study comprising 1457 patients with AP, triglycerides, utilized as a quantitative variable in 100 mg/dL increments, demonstrated independent associations with organ failure, pancreatic necrosis, acute toxicity, and mortality.^[22]^ Our results align with this pattern; however, in models B and C, no significant difference in ICU hospitalization rates was observed between the group with moderately elevated blood triglyceride levels and the normal lipid group. We postulate that this lack of significance may be attributed to factors such as clinician decision-making, extent of pancreatic injury, and other related variables. High TG levels have received considerable attention from healthcare professionals; however, moderately elevated TG levels may not necessarily result in significant damage to the pancreas.

The association between HTG and AP onset lacks a clearly defined threshold. However, the risk of developing AP gradually increases with increasing triglyceride levels.^[23]^ The pathophysiology of HTG-induced AP involves accumulation of free fatty acids (FFAs) and subsequent activation of the inflammatory response. The exceptionally high concentrations of FFAs exceeded the binding capacity of plasma albumin. Consequently, FFAs self-aggregate and form micellar structures with detergent properties that cause damage to platelets, acinar cells, and vascular endothelial cells, initiating a cascade of pancreatic injuries.^[24]^ The severity of pancreatitis is contingent upon the intensity of the inflammatory response and the extent of lipotoxicity-induced damage.^[23]^ According to experimental animal models, a specific study has shown that the presence of HTG can aggravate pancreatic injury in the context of AP.^[25]^ Additionally, Yang et al discovered that the coexistence of HTG and obesity increases the severity and incidence of local complications in AP, with HTG playing a substantial role in the risk of comorbidity.^[26]^

In the United States, disparities in early mortality rates exist among different ethnic groups owing to variations in race, particularly influenced by socioeconomic status.^[27]^ One study indicated that black patients have a higher hospitalization rate for AP and are more susceptible to alcohol-induced AP.^[28]^ An epidemiological study conducted from 2001 to 2014 found that acute pancreatitis with hypertriglyceridemia was more prevalent among males, Hispanics, individuals aged 35 to 44 years, those with private insurance, and in facilities located in the Western United States.^[29]^ In our subgroup analysis, we observed a higher likelihood of ICU admission in nonwhite individuals with HTG, which is potentially attributable to factors such as higher poverty rates, limited access to medical resources, and dietary habits. Notably, our findings revealed elevated rates of ICU admission in individuals without hypertension. In patients with AP, especially severe AP, the pancreas releases large amounts of pro-inflammatory cytokines, leading to a systemic inflammatory response syndrome and severe intravascular fluid loss.^[30]^ This can cause mixed hypovolemia and distributive shock, ultimately leading to multi-organ failure.^[31,32]^ This could be the reason for it.

Currently, despite the availability of effective treatments such as insulin, heparin, plasmapheresis, and anti-HTG drugs for patients with HTG-AP, there is a lack of established guidelines for its treatment.^[4]^ The current approach to managing HTG-AP involves initiating drug therapy to rapidly and consistently lower TG levels to below 5.65 mmol/L.^[33]^ Ozcelik et al conducted a study demonstrating the safety and efficacy of combination therapy using insulin, heparin, and fenofibrate.^[34]^ Another strategy for reducing TG levels involves adopting a healthy lifestyle, particularly following the dietary pattern of the Mediterranean diet.^[35,36]^ Therapeutic plasma exchange (TPE) is a safe, prompt, and effective treatment modality.^[37]^ Early implementation of TPE is a reliable and independent approach to reduce plasma TG levels in HTG-AP,^[38]^ regardless of the occurrence and duration of organ failure in acute pancreatitis.^[39]^

Nevertheless, our study has certain limitations. Primarily, it is crucial to acknowledge that this retrospective cohort study relied on data from the MIMIC-IV database, constituting a single-center dataset and potentially introducing a selection bias. Despite a thorough adjustment for potential confounders, the association between TG levels and ICU admission may still be influenced by unmeasured confounding factors. In addition, the exclusion of patients with cirrhosis, malignant tumors, or AIDS limits the generalizability of our findings to these specific populations. Therefore, validation and reinforcement of these results necessitate large-scale multicenter studies.

## 5. Conclusions

In conclusion, a notable linear relationship was observed between ICU admission and blood triglyceride levels. As triglyceride levels increased, there was a corresponding increase in ICU admission. Patients within the 1.7–5.65 mmol/L and > 11.3 mmol/L triglyceride groups exhibited higher rates of ICU admissions. Moreover, we observed a higher risk of ICU hospitalization even with mild TG elevation.

## Author contributions

**Conceptualization:** Shuaiyong Wen.

**Data curation:** Shuaiyong Wen.

**Formal analysis:** Shuaiyong Wen.

**Funding acquisition:** Yunfeng Cui.

**Investigation:** Shuaiyong Wen.

**Methodology:** Shuaiyong Wen.

**Project administration:** Yunfeng Cui.

**Resources:** Yunfeng Cui.

**Software:** Shuaiyong Wen.

**Supervision:** Yunfeng Cui.

**Validation:** Yunfeng Cui.

**Writing – original draft:** Shuaiyong Wen, Yu Zhang, Guijie Zhao.

**Writing – review & editing:** Kun Zhang, Zhengwei Tu, Yunfeng Cui.
